# Characterizing SARS-CoV-2 viral clearance kinetics to improve the design of antiviral pharmacometric studies

**DOI:** 10.1128/aac.00192-22

**Published:** 2022-07-19

**Authors:** James A Watson, Stephen Kissler, Nicholas PJ Day, Yonatan Grad, Nicholas J White

**Affiliations:** 1 Mahidol-Oxford Tropical Medicine Research Unit, Faculty of Tropical Medicine, Mahidol University, Bangkok, 10400, Thailand; 2 Centre for Tropical Medicine and Global Health, Nuffield Department of Medicine, University of Oxford, New Richards Building, Old Road Campus, Roosevelt Drive, Oxford, OX3 7LG, UK; 3 Department of Immunology and Infectious Diseases, Harvard T. H. Chan School of Public Health, Boston, MA 02115, USA

**Keywords:** SARS-CoV-2, Phase 2, Antiviral drugs, Pharmacodynamics, Clearance rate, Time to clearance

## Abstract

A consensus methodology for the pharmacometric assessment of candidate SARS-CoV-2 antiviral drugs would be useful for comparing trial results and improving trial design. The time to viral clearance, assessed by serial qPCR of nasopharyngeal swab samples, has been the most widely reported measure of virological response in clinical trials, but it has not been compared formally with other metrics, notably model-based estimates of the rate of viral clearance. We analyzed prospectively gathered viral clearance profiles from 280 infection episodes in vaccinated and unvaccinated individuals. We fitted different phenomenological pharmacodynamic models (single exponential decay, bi-exponential, penalized splines) and found that the clearance rate, estimated from a mixed effects single exponential decay model, is a robust pharmacodynamic summary of viral clearance. The rate of viral clearance, estimated from viral densities during the first week following peak viral load, provides increased statistical power (reduced type 2 error) compared with time to clearance. Antiviral effects approximately equivalent to those with currently used and recommended SARS-CoV-2 antiviral treatments, notably nirmatrelvir and molnupiravir, can be detected from randomized trials with sample sizes of only 35 to 65 patients per arm. We recommend that pharmacometric antiviral assessments should be conducted in early COVID-19 illness with serial qPCR samples taken over one week.

## Introduction

Acute SARS-CoV-2 infection can be characterized approximately as two overlapping clinical stages. The first pre-symptomatic stage comprises uncontrolled viral replication. Peak viral loads in the nasopharynx or oropharynx of individuals with symptomatic COVID-19 illness occur around the time of symptom onset (1). The second stage of infection comprises a first order decrease in the viral load resulting from activation of host-defense mechanisms. Viral multiplication is attenuated, and clearance is augmented by effective host defenses (2). During this second stage a small subset of infected individuals (<5%) progress to severe pneumonia and some will die. The risk is strongly age dependent (3) and it is reduced substantially by vaccination (4). Infections with the now-prevalent Omicron variant are associated with a lower risk of hospitalization.

Effective antiviral drugs or biologics attenuate viral multiplication (5, 6). Antiviral interventions are most effective early in the course of disease, while immunomodulators are life-saving in hospitalized patients as immunopathology dominates after approximately one week of illness (7). Effective antiviral medicines administered early during SARS-CoV-2 infections should reduce the overall viral load, accelerate virus clearance, and reduce the probability of progression to severe COVID-19 illness. These beneficial effects have been shown most convincingly for the monoclonal antibody mixture of casirivimab and imdevimab (REGN-CoV-2) (5), and recently for the ribonucleoside analogue molnupiravir (6, 8) and the main protease inhibitor nirmatrelvir (9).

Primary end-points in hospital based randomized controlled trials (RCTs) have usually been either the need for respiratory support or death whereas, in outpatient studies, measures such as changes in symptom severity or evidence of clinical progression (e.g. SpO_2_ <92%) are used generally (10–12). Large sample sizes are required to detect meaningful effects in outpatient studies, even if high risk subgroups only are enrolled, as only a minority of symptomatic individuals develop hypoxemia and progress to require hospitalization (10). In vaccinated populations progression to severe disease is now unusual. How then can the long list of repurposed candidate antivirals be prioritized for phase 3 evaluation, and how can the expanding number of candidate new antivirals be compared? Accurate measurement of viral clearance is potentially the most efficient method of ‘phase 2’ candidate drug screening and dose-finding, but there is currently no consensus on the pharmacometric methodology.

In the first week after becoming symptomatic with COVID-19, viral concentrations in nasopharyngeal or oropharyngeal swab samples decline exponentially (13, 14). It is increasingly evident that the pattern of viral elimination is often bi-exponential (15), although the second phase, which is close to the limit of accurate viral quantitation, may be unobserved if follow-up is not extended. In the context of a clinical trial viral clearance can be summarized in many different ways. The most commonly reported is time-to-clearance (the time to reach the lower limit of detection for a given assay, see for example (12)) but this is dependent on both the assay used and the baseline viral load (i.e. the viral load at enrolment in the study, which will depend on how cases are ascertained and the individual peak viral loads which vary substantially across individuals). We used prospectively gathered data from 280 infection episodes from individuals who had frequent qPCR sampling before and after peak viral load to characterize the kinetics of viral clearance and to propose robust summary statistics as pharmacodynamic endpoints for pharmacometric evaluations and phase 2 type clinical trials.

## Materials and Methods

### Data

We obtained prospective longitudinal SARS-CoV-2 RT-qPCR testing data on 474 infection episodes from individuals in the US National Basketball Association’s (NBA) occupational health program, observed between November 28 2020 and January 10, 2022. These infection data have been described previously in detail (2, 13, 16). The data are openly accessible on github at https://github.com/gradlab/CtTrajectories_AllVariants and https://github.com/gradlab/CtTrajectories_Omicron. In brief, clinical samples were obtained by combined swabs of the anterior nares and oropharynx for each individual administered by a trained provider. Viral load was measured from cycle threshold (CT) values measured in the Roche cobas target 1 assay. The CT values were transformed to log_10_ RNA copies per mL using data from synthetic controls (intercept: 11.3408917; slope: -0.2770306) (13). Vaccination information was reported and verified by NBA staff and the clinical operational team.

### Data pre-processing

For each infection episode, we initially set day 0 as the day with the lowest observed CT value (i.e. highest nasopharyngeal or oropharyngeal viral loads). We selected all infection episodes where there were at least 5 samples with a CT value less than 40 and at least one sample with a CT value less than 30 in the time period -20 to +20 days for each individual (a total of 280 infections). To these infection data, we then fitted a simple Bayesian hierarchical ‘up-and-down’ phenomenological model to the log_10_ RNA copies per mL values (14). The model was specified as:

Equation 1 $$f_{vl}\left(t\right)=A_{0}+\text{log}\left(\frac{x+y}{ye^{-x\left(t-t_{\max}\right)}+xe^{y\left(t-t_{\max}\right)}}\right)$$

where *A_o_* corresponds to the peak viral load occurring at time *t = t_max_* (i.e. the intercept); *x* is the growth rate in the initial viral proliferation stage; and *y* is the decay rate in the viral clearance stage. The main purpose of this model was to estimate the time of peak viral load (*t_max_*) for each individual. Individual random effect terms with a joint covariance structure were specified for *A*_0_, *x*, *y* and *t_max_* (all additive random effects). For each individual we then defined time zero as their mean estimated *t_max_*. We then selected all viral load measurements taken during the interval [*t_max_*, *t_max_* + 14]. This gave a total of 280 infection episodes with a median of 7 viral load measurements above the lower limit of detection post peak viral load per episode (range: 1 to 18).

### Model of viral clearance dynamics

To characterize the viral clearance in these 280 infection episodes, we fitted a series of phenomenological models. We chose not to fit mechanistic models (e.g. (17)) as the goal was not explain the biology underlying viral clearance but to provide a robust method from which to infer summary statistics for virological clinical trial endpoints.

#### Model 1: Exponential decay

The simplest model for viral clearance is log-linear decay specified as:

Equation 2 $$f_{vl}\left(t\right)=A_{0}-\alpha t$$

where *A*_0_ is the peak log viral load (intercept at *t* = 0) and *α* is the clearance rate.

#### Model 2: Bi-exponential decay

There is evidence of persistent viral shedding at low densities (<1,000 RNA copies per mL) (13). Bi-exponential decay (also known as a “two-compartment” model) can fit this type of clearance profile, and is defined as:

Equation 3 $$f_{vl}\left(t\right)=\text{log}\left(e^{A_{0}-\alpha t}+e^{B_{0}-\beta t}\right)$$

where *B*_0_ < *A*_0_ and β < α. Under this model, the intercept at time *t* = 0 is log (*e*^*B*_0_^ + *e*^*A*_0_^ (which is approximately equal to *A*_0_ if *B*_0_ << *A*_0_); *α* is the rate constant for the initial ‘fast’ decay; and *β* is the rate constant for the second ‘persistence’ stage.

#### Model 3: Regularized splines

We used penalized linear models from the R package *mgcv* which implements penalized regression splines with automatic smoothness estimation (18). We specified random effect terms for each infection episode.

#### Error model for models 1-2

It was been observed previously that there is considerable variation in viral load estimates from oropharyngeal and nasopharyngeal swabs (13, 14). This can result from several factors including variations in local viral densities, in swab size and swabbing technique, number of cells, volume of eluate, and qPCR assay variability. To account for these different sources of variation, we used robust linear regression with a t-distribution error model (19) with unknown degrees of freedom *k* inferred from the data.

#### Hierarchical model and prior distributions

The data pre-processing model (equation 1: ‘up-down model’) and the main clearance models 1 and 2 were specified as hierarchical Bayesian models with correlated random effect terms for the slope coefficient(s) and the intercept(s). For infection *i* for model 1 this was parameterised as:

$$f_{vl}^{i}\left(t\right)=\left(A_{0}+\theta_{i,1}\right)+\left(\alpha e^{\theta_{i,2}}\right)t$$

The first random effect *θ*_*i*,1_ represents an individual shift from the population intercept *A*_0_ (giving the individual log peak viral load); the second random effect *θ*_*i*,2_ represents the log of the proportional increase or decrease in the population rate constant *β*.

For model 2 the random effects were parameterized as:

$$f_{vl}^{i}\left(t\right)=\text{log}\left(e^{A_{0}+\theta_{i,1}-\left(\alpha e^{\theta_{i,2}}\right)t}+e^{B_{0}+\theta_{i,3}-\left(\beta e^{\theta_{i,4}}\right)t}\right)$$

where *θ_i,k_, k* = 1..4 have analogous interpretations as in model 1.

We specified weakly informative priors for all parameters of the two Bayesian hierarchical models. The prior values were informed by previous analyses (13):

$$\begin{array}{c} A_{0}∼\text{Normal}\left(5,2\right)\\ B_{0}∼\text{Normal}\left(3,2\right)\\ \alpha ∼\text{Normal}\left(0.5,1\right)\\ \beta ∼\text{Normal}\left(0.1,1\right)\\ k∼\text{Exponential}\left(1\right)\\ \theta ∼\text{Normal}\left(0,\Sigma \right) \end{array}$$

For *A*_0_ (the intercept), the prior corresponds to a population mean peak viral load of approximately 10^5^ RNA copies per mL. For *B*_0_ intercept this corresponds to the persistent viral shedding phase starting at around 1,000 copies per mL. The variance-covariance matrix *∑* was parameterised as its Cholesky LKJ decomposition, where the L correlation matrix had a uniform prior (i.e. hyperparameter *v*=1). We ran a sensitivity analysis with quasi-flat priors (all standard deviation values multiplied by 10), see supplementary materials.

### Clinical trial simulation and power analysis

We used the posterior predictive distribution under model 2 (bi-exponential decay) to simulate clinical trial data under the assumption that patients were enrolled approximately at the time of their peak viral load. The data simulation algorithm for each clinical trial dataset of size *N* was specified as follows:

- Draw a single posterior sample *θ* from model 2 fit to the clearance data: *θ* determines the population clearance rates *α, β* and the population intercepts *A*_0_, *B*_0_, and the variance-covariance matrix from which we generate a matrix of random effects for each patient under model 2 (equation 3, random effects are of size *N*-by-4).
- For each individual *i* = 1..*N* we generate their serial viral load data as follows:
  a. With probability *p* the patient’s peak viral load occurs before enrolment (*p* = 0.8 in the main simulations), the time since peak is then drawn from a uniform random distribution over [0,4] days; with probability 1 − *p* the peak occurs after enrolment: the time until peak is drawn from a uniform random distribution over [0,2] days;
  b. For viral load observations taken before the peak viral load: generate increasing viral loads using a fixed coefficient of 1.5 (under the ‘up-down’ model given in equation 1). For viral load observations taken after the peak, decreasing viral loads are generated according to the bi-exponential decay model (equation 3).
  c. For the patients randomized to the treatment arm, the individual decay rate coefficients *α_i_*, *β_i_* are multiplied by the treatment effect (either 1.3 or 1.5, or 1 in the null simulations).
  d. Additive measurement error is then added using independent and identically distributed random draws from a *t*-distribution with mean 0 and degrees of freedom and variance defined by *θ*.

In each simulation we assumed that the clinical trial was comparing no intervention to an antiviral agent whose effect size *γ* was parameterised on the log scale as a constant scaling factor on the population mean rate coefficients *α* and *β*. We simulated trials that did once or twice daily swabs (performed independently) over either 5 or 7 days (between 6 and 16 viral load measurements). The treatment effects used in the simulations (1.5 and 1.3, corresponding to 50% and 30% increases in the slope of viral clearance) were chosen from visual inspection of the published viral load data after treatment with the casirivimab-imdevimab (Regeneron) monoclonal antibody in pre-Omicron infections (5), nirmatrelvir+ritonavir (9), and molnupiravir (6). For all three treatments, published viral load data were presented as group means over time, and the increases in slope in the mean viral loads are between 30 and 50% approximately.

For each simulated dataset we then estimated the treatment effect by fitting the single exponential model (equation 2, thus mis-specified by design). The treatment effect *γ* was then estimated from the simulated data under the log-linear model (equation 2), which is also parameterised on the log scale representing a proportional change in the population clearance rate:

$$f_{vl}^{i}\left(t,Z_{i}\right)=\left(A_{0}+\theta_{i,1}\right)+\left(\alpha e^{\gamma 1\left\{Z_{i}=1\right\}+\theta_{i,2}}\right)t$$

where *Z_i_* is the randomised treatment allocation (1 is treated, 0 not treated) and 1 is the indicator function taking value 1 if *Z_i_* and 0 otherwise. We gave *γ* a Normal (0,1) prior. If the 2.5% percentile of the posterior distribution over *γ* was greater than 0, this was defined as rejecting the hypothesis that the intervention had no effect (i.e. a one-sided test at the 2.5% level).

For comparison, we estimated Kaplan-Meier survival curves for the time to clearance in each treatment arm, whereby a clearance event was defined as both the qPCR swabs on a given day being equal or lower than the lower limit of detection (equivalent to a CT value of 40). If the median time to clearance in the treated group was shorter than the median time to clearance in control group we then tested for a difference between the survival curves using the log-rank test. Rejection of the null hypothesis was defined as a p-value <0.025 (also a one-sided test at the 2.5% level). For both the rate of clearance and time to clearance simulations, the power was defined as the proportion of simulations which correctly rejected the null hypothesis of no treatment effect. To check the correctness of the algorithm we also simulated data under no treatment effect (no acceleration in viral clearance, *γ* = 0) to verify that the type 1 error approximates 2.5%.

## Statistical analysis

### All statistical analyses were performed in R version 4.0.2

We defined the time to clearance as the time to the first recorded CT value equal to 40, or with right censoring at the last recorded time point if all values were less than 40. We compared the survival curves of the COVID-19 vaccinated individuals and the unvaccinated individuals using the log-rank test (as implemented in the R package *survival*, function *survdiff*).

For all Bayesian models of viral clearance, we considered viral loads equivalent to a CT value of 40 as left censored. The likelihood of the model parameters *θ* given the data is thus equal to $\int_{-\infty }^{LOD}f(x|\theta ),$, where *f*(. |θ) is the model likelihood and LOD is the lower limit of detection.

Bayesian hierarchical models were coded in *stan* and fitted using Hamiltonian Monte Carlo with the *rstan* package (20). For model 2, to avoid label switching across the parameters *A*_0_, *B*_0_ and *α, β*, we used the *ordered* vector type. Penalized additive linear models were fitted using the R package *mgcv* (18). We checked convergence of chains by visual inspection of trace plots. All data and code are available at https://github.com/jwatowatson/SARS-CoV2-Viral-Clearance-Kinetics.

## Results

### Phenomenological models of viral clearance

**Figure 1:**
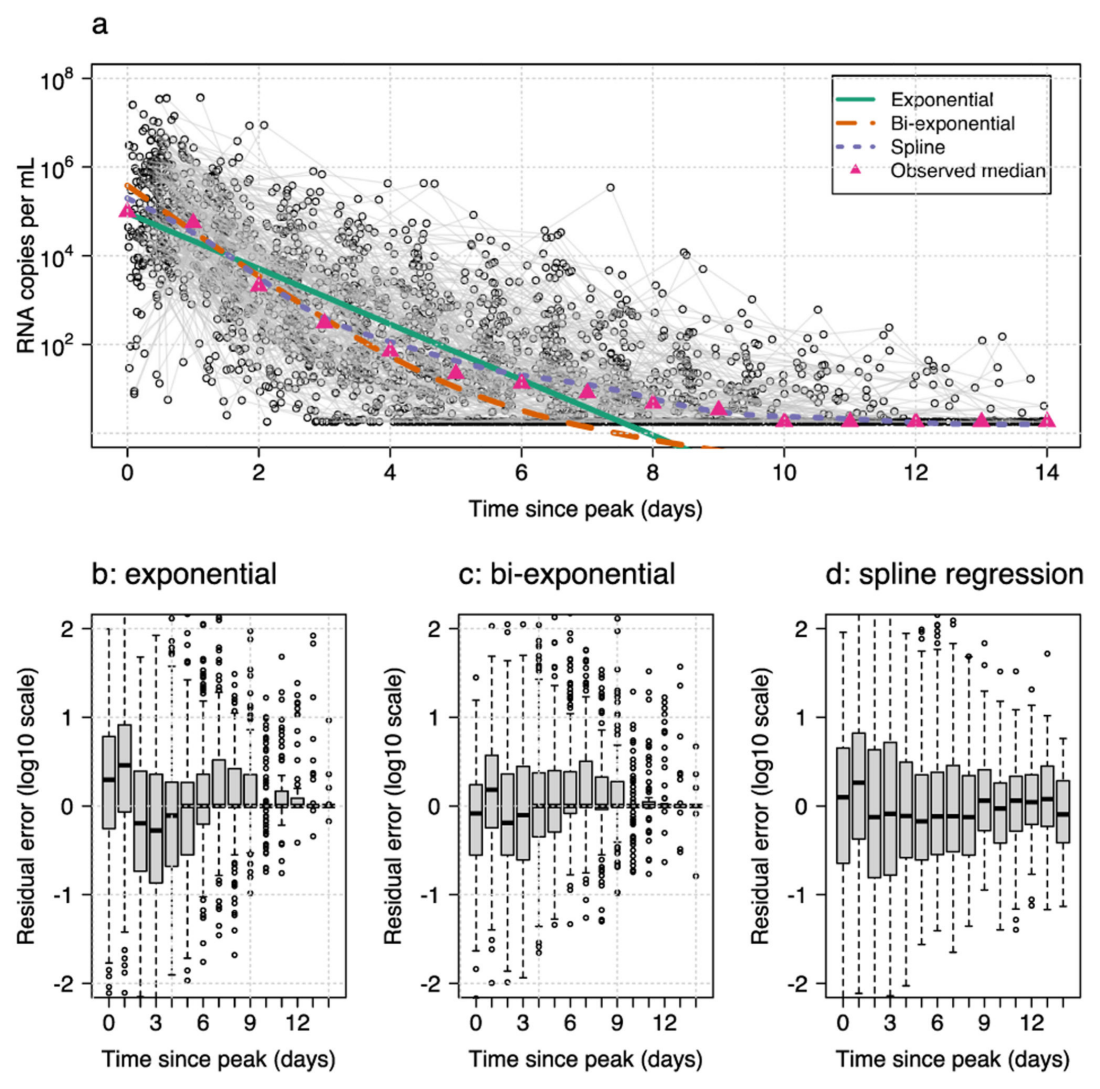
Comparing model fits for three phenomenological models of SARS-CoV-2 viral clearance. **a** Spaghetti plot of the data is shown with the mean predicted values from the three models (exponential: green; bi-exponential: orange; penalized splines: purple). The daily median viral loads are shown by the pink triangles. **b-d** Distribution of the residuals as a function of time since peak viral load.

After data pre-processing we had serial viral load data after the inferred peak viral load for 280 infection episodes with a median of 8 samples per infection (Figure 1). We fitted three phenomenological models to these post-peak clearance data: a single rate exponential decay Bayesian hierarchical model (equation 2); a bi-exponential decay Bayesian hierarchical model (equation 3); and a random effects additive linear model (penalized spline regression). All three models showed some bias in the distribution of the residuals as a function of the time since peak viral load, with the strongest bias appearing for the single exponential decay model. As the overall profile of viral clearance is clearly non-linear (as demonstrated by the daily median log_10_ viral loads shown by the pink triangles in Figure 1a, note that the median is unbiased when fewer than half the values are left censored), the linear model gives a compromise fit with a lower intercept and shallower slope. The bi-exponential decay model had a substantially better fit with less bias in the residuals as a function of time since peak. This was mostly due to a subset of infections which clearly demonstrated a second phase (see individual fits in supplementary Appendix). The penalized spline regression model did not give an improved fit compared to the bi-exponential model with larger residual errors.

### Pharmacodynamic summaries of viral clearance

For a single exponential decay model (model 1), viral clearance of the *i^th^* infection can be summarized by the estimated individual clearance rate *βe*^*θ*_*i*,2_^. However, for a bi-exponential decay model (model 2), there are two individual clearance rates *βe^θ_i,2_^* and *γe*^*θ*_*i*,4_^ and an additional ‘elbow’ intercept *B_0_ + *θ*_*i*,3_*, thus giving three partial summaries of the viral clearance. These can be combined into a single metric by taking the area under the curve (AUC) of the estimated log viral load with left censoring at the lower limit of detection. In order for the AUC to be independent of the baseline viral load, it is necessary to scale the AUC by the estimated intercept. Note that for the single exponential model the AUC is directly proportional to the clearance rate.

**Figure 2:**
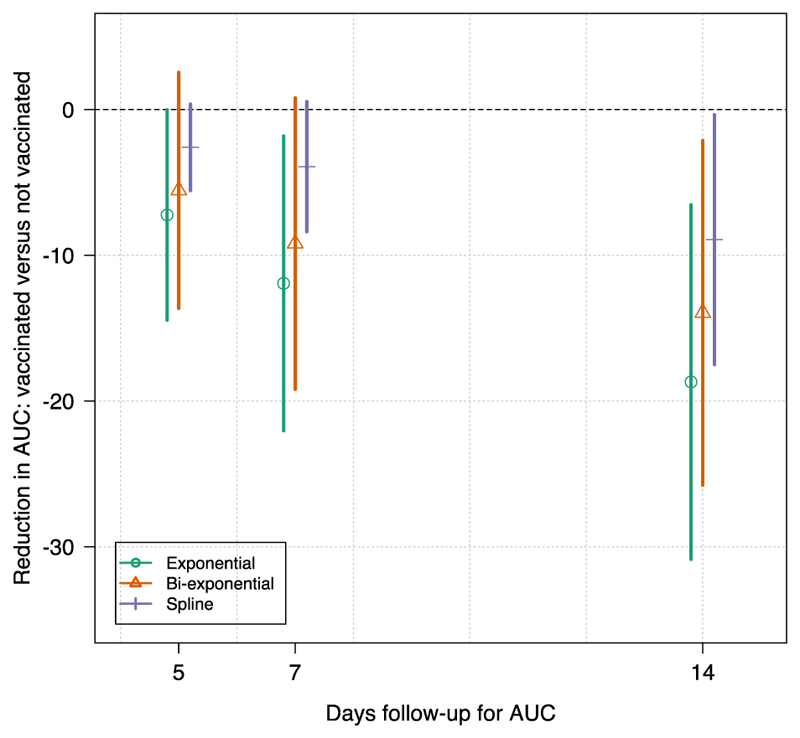
Differences in AUC for the estimated ΔCT in infection episodes in vaccinated (n=17) and unvaccinated (n=60) individuals. The estimated AUC is scaled by the intercept to remove the dependence on the baseline viral load. Approximate confidence intervals are calculated from a t-test.

Out of the 280 infection episodes, 77 had a confirmed vaccination status whereby 17 infections occurred in fully vaccinated individuals and 60 in non-vaccinated individuals. Consistent with the previously published analysis of the same dataset (2) and other separate data (14), the single exponential model suggested a faster clearance rate in the vaccinated compared with the unvaccinated subjects (p=0.05 comparing random effect terms for the slope coefficient *α*). As measured by the AUC estimated until day 5, day 7, or day 14, the log-linear model estimated larger reductions in AUC in vaccinated relative to unvaccinated individuals than either the bi-exponential or the spline regression models (Figure 2).

**Figure 3:**
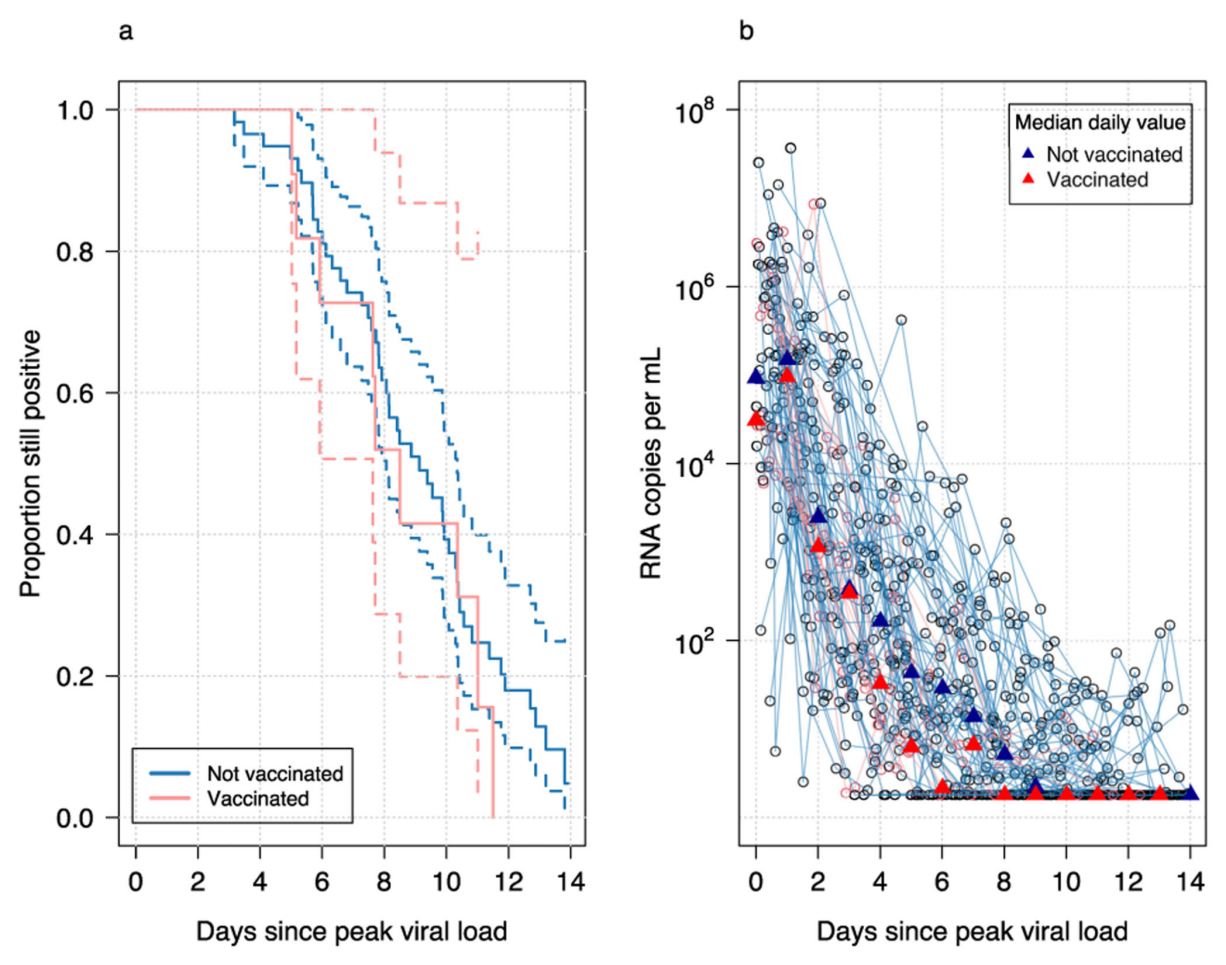
Times to viral clearance in vaccinated and unvaccinated individuals. **a** Kaplan-Meier survival curves (with 95% confidence intervals) of the proportion still testing positive over time. **b** Individual viral load profiles with daily median values shown by the triangles. Pink: vaccinated; dark blue: unvaccinated.

The time to viral clearance is the virological endpoint most commonly reported in COVID-19 clinical trials (a model-free measure, although the exact definition of this measure varies across trials). Time to clearance as a summary statistic is ‘inefficient’ because it is strongly dependent on the initial viral load; it does not borrow information across time points; and it only uses the noisy estimate of when the viral load first reaches the lower limit of detection. It is also influenced by the variable contribution of the second slower phase of viral clearance, and is dependent on the assay sensitivity. In contrast to rate of clearance there was no difference between time to clearance in the vaccinated versus unvaccinated individuals (Figure 3; p=0.2 for a log-rank test).

### Sample size estimation

**Figure 4:**
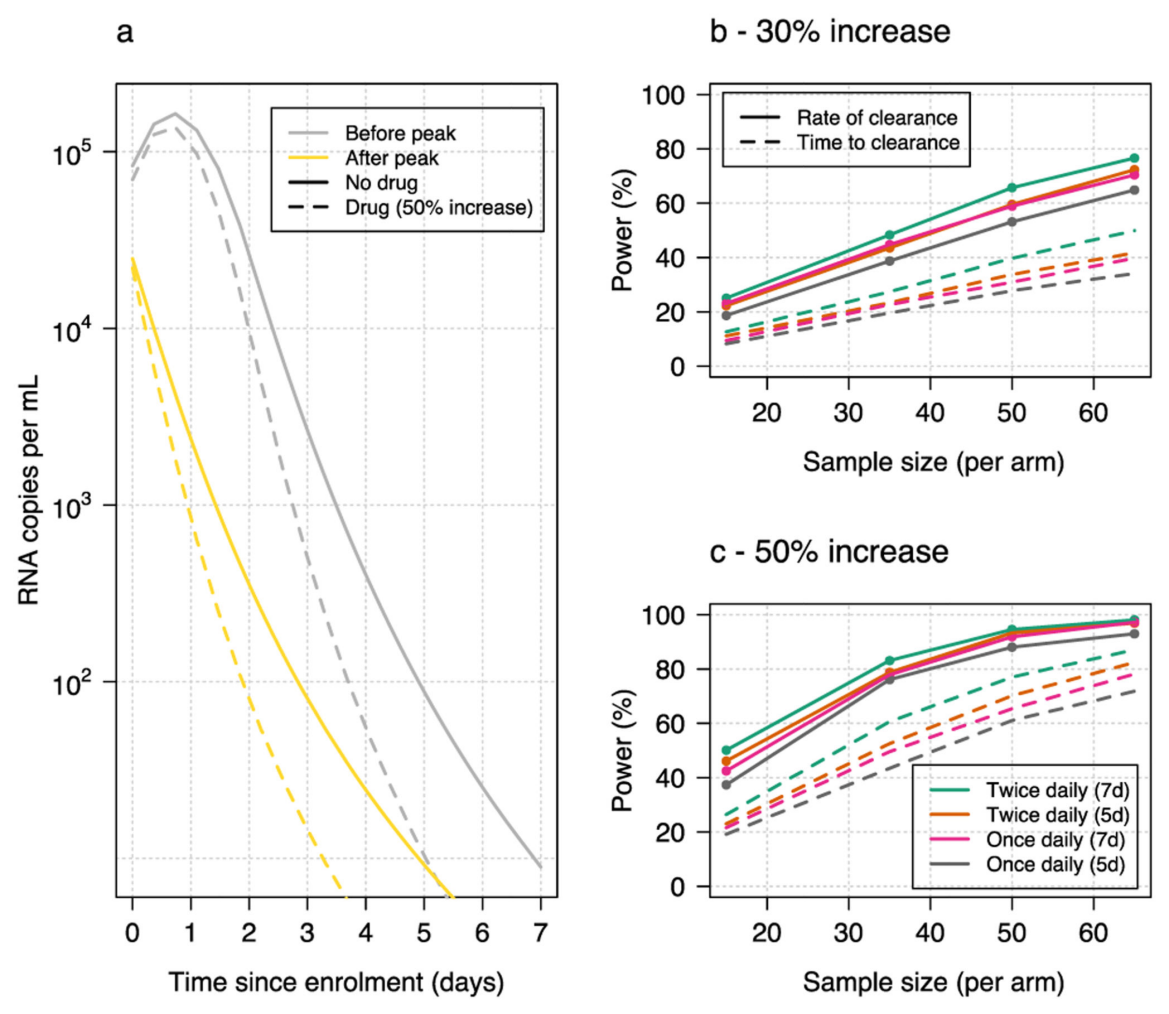
Power calculations for time to clearance or rate of clearance based on simulated clinical trial data. **a** Median viral load (log_10_ scale) over time for the simulated data (20% of patients are recruited before their peak, shown in grey; 80% of patients are recruited after their peak, shown in yellow). The thick lines show the median clearance profile under no intervention; the dashed lines show the median clearance profile for interventions with effect sizes of 50% (the effect size is defined as the proportional increase in rate coefficients α, β in the data generating model). **b-c** Estimated power (1 - type 2 error) when comparing rates of clearance (thick lines) under a single exponential model or time to clearance (dashed lines) for four sampling schemes (once or twice daily for 5 or 7 days).

Previous work has estimated that sample sizes of approximately 500 patients per arm are required for clinical trials evaluating differences in time to viral clearance as primary end-points (12). We postulated that by estimating the rate of clearance directly from frequent serial viral load measurements, necessary sample sizes could be reduced substantially. By estimating the rate of clearance using a robust linear model information is borrowed across the serial viral load measurements, thereby resulting in a gain in efficiency relative to the standard approach of estimating the time to clearance. To explore this, we simulated patient data under the following assumptions: 80% of patients are recruited within 4 days of their peak viral load and the viral load follows a bi-exponential decay (simulated using the posterior predictive distribution of model 2 fitted to the NBA dataset, shown by the yellow lines in Figure 4a); the remaining 20% of patients are recruited before their peak viral load with a time until peak of up to 2 days (shown by the grey lines in Figure 4a). For the patients recruited before their peak viral load the initial increase is modelled as per equation 1, followed by bi-exponential decay. We assumed effect sizes equal to 30% and 50% increases in the population viral clearance rate parameters of the model. Figure 4a shows the median viral load values over time with no antiviral treatment (thick lines) and with treatment (dashed lines shows the decay for an intervention with 50% increase in slope).

We used these simulated data to estimate the power of a clinical trial with 1:1 randomization between no treatment and an intervention with a treatment effect of either 30% or 50% increase in viral clearance. In this the null hypothesis of no treatment effect was either tested by modelling clearance rates directly (under a single exponential model, thus mis-specified by design) or by comparing survival curves for the time to clearance across the two groups. Figure 4b-c shows the estimated power (1 - type 2 error) for sample sizes between 15 and 65 patients per arm, with either once or twice daily swabbing for 5 or 7 days. In all simulations, greater power was obtained by modelling the rate of clearance directly even though this was done under a mis-specified model (mis-specification is not an issue for time to clearance, which is a model free statistic). The average increase in power was over 20 percentage points. These simulations suggest that over 80% power for a type 1 error rate of 2.5% can be obtained with only 35-65 patients per study arm if samples are taken twice daily over 7 days in early illness.

## Discussion

An effective, well tolerated, safe, affordable and generally available treatment of COVID-19 that prevented progression to severe disease would be of enormous global health benefit. There have been many small clinical trials assessing the efficacy of repurposed candidate antivirals, justified usually by moderate inhibitory activity in virus cell cultures, but actionable evidence has come mainly from relatively few large randomized controlled trials in hospitalized patients. However, by the time patients require hospital care, viral loads have declined and immune pathology dominates the clinical picture. In these large RCTs immunomodulators (notably corticosteroids and IL-6 antagonists) have proved life-saving (7, 21). Recently, large RCTs in outpatients with uncomplicated COVID-19, mainly comprising “high risk” individuals (i.e. older patients and those with co-morbidities predisposing to more severe illness), have shown significant clinical benefits with both SARS-CoV-2 directed monoclonal antibodies (5) and small molecule antiviral drugs; notably the nucleoside analogue molnupiravir (6), and the ritonavir boosted viral protease inhibitor nirmatrelvir (9). Early administration of remdesivir has also been shown to reduce hospitalizations (22). Apart from these, there are a large number of both repurposed and novel antiviral drugs either in development or under consideration for COVID-19 prophylaxis or treatment, but there is no agreed methodology for testing them in vivo or for determining the optimum dosage. It is simply not possible to conduct very large RCTs on each potential antiviral treatment, so a method of in-vivo pharmacometric assessment is needed in order to choose potential candidates for larger “phase 3” evaluations, to compare new antiviral therapies, and to optimize dosing. Although COVID-19 is a systemic infection, viral clearance from the upper respiratory tract is the only readily accessible measure of an ambulant patient’s virological response. Viral densities in nasopharyngeal or oropharyngeal swab samples peak around the time of clinical presentation and then exhibit a bi-exponential decline within the first 7-10 days of illness. Previous vaccination or infection accelerates viral clearance (2, 14). There is substantial variation between individuals, but mean profiles in untreated or placebo treated outpatients are very similar across large studies.

Our analyses suggest that viral clearance is best assessed by measuring the approximate slope of the log-linear decline in qPCR densities over the first week after starting treatment accounting for left-censoring. In many patients a bi-phasic elimination profile is evident, but the majority of viral load reduction is in the initial phase, and it is this phase which can be accelerated by effective interventions (vaccination, monoclonal antibodies, small molecule antivirals). Although mis-specified, the log-linear model appears to capture in a robust way the overall trend of viral clearance. The widely reported time to viral clearance is a less precise measure, and it results in lower statistical power in pharmacometric studies. This is because the time to viral clearance depends on the frequency of testing, the sensitivity and reproducibility of the method and, critically, the initial virus density (and thus the stage of disease). It is also variably confounded by detection of the slower terminal virus elimination phase. An alternative is to use mechanistic models of viral kinetics but it is unclear whether this would have an advantage over robust linear regression given that the underlying viral dynamics are likely to be highly complex (23).

Viral clearance reflects both host-defense and any additional contribution from an effective antiviral therapeutic. Thus, the pattern of clearance will depend on the stage of disease and the host’s response. We propose using the slope of the initial log-linear decline in viral densities in nasopharyngeal or oropharyngeal secretions (i.e., the rate constant of the major decline) as the primary endpoint in phase 2 studies. The difference between the slopes with and without the putative antiviral represents the drug effect. Evaluations of therapeutic interventions which do not report these values and use time to viral clearance as the primary endpoint are difficult to interpret. Time-to-clearance results in a higher type 2 error (lower power) than rate-of-clearance, and therefore requires larger sample sizes, at least daily sampling, and a longer duration of follow-up. It is therefore imprecise, inefficient, and consequently expensive. Importantly, any comparison or meta-analysis of studies which used different sampling techniques, or had different qPCR sensitivities, or different cut-offs, is confounded systematically if time-to-clearance is used as an endpoint, but not if rate-of-clearance is measured. It is important to note that comparisons between interventions should be contemporary as both immune status and viral variants are important contributors to viral clearance. Observational studies using historical controls would likely give misleading results.

There are several limitations to this study. The data were obtained from a monitoring scheme and not a prospective study of antiviral pharmacodynamics. The COVID-19 pandemic has evolved rapidly with an increasing proportion of the population being immunized and rapid changes in viral variants. More information on viral dynamics in infections with the Omicron variant in vaccinated individuals is needed. The rate constant of the decline in viral densities over one week is a hybrid parameter, and more information is required on virus clearance profiles in different settings to determine if this is the optimum pharmacodynamic measure in all circumstances. There is substantial variation in measured viral densities, even within an individual infection, and more information on the sources of variance is needed to refine these approaches.

In summary our model-based simulations suggest that pharmacometric assessment of candidate antivirals for COVID-19 should measure virus clearance rate, and not the much more widely used time to clearance, as their primary endpoint. If performed in early uncomplicated illness, reasonable precision can be obtained with twice daily qPCR samples taken over the course of one week after enrolment in each studied patient. Adaptive randomization using the viral clearance rate can thereby rapidly identify active intervention arms.

## Supplementary Material

Supplementary Appendix
