# Supplementary material for: Characterizing SARS-CoV-2 viral clearance kinetics to improve the design of antiviral pharmacometric studies
Source: Antimicrob Agents Chemother. Author manuscript; Available in PMC 2022 Jul 23. (PMC9295592; doi:10.1128/aac.00192-22)
Supplement: Supplementary Appendix [file EMS146347-supplement-Supplementary_Appendix.pdf]

Supplementary Materials: Characterising SARS-CoV-2 viral clearance kinetics to improve the design of antiviral pharmacometric studies

Parameter estimates

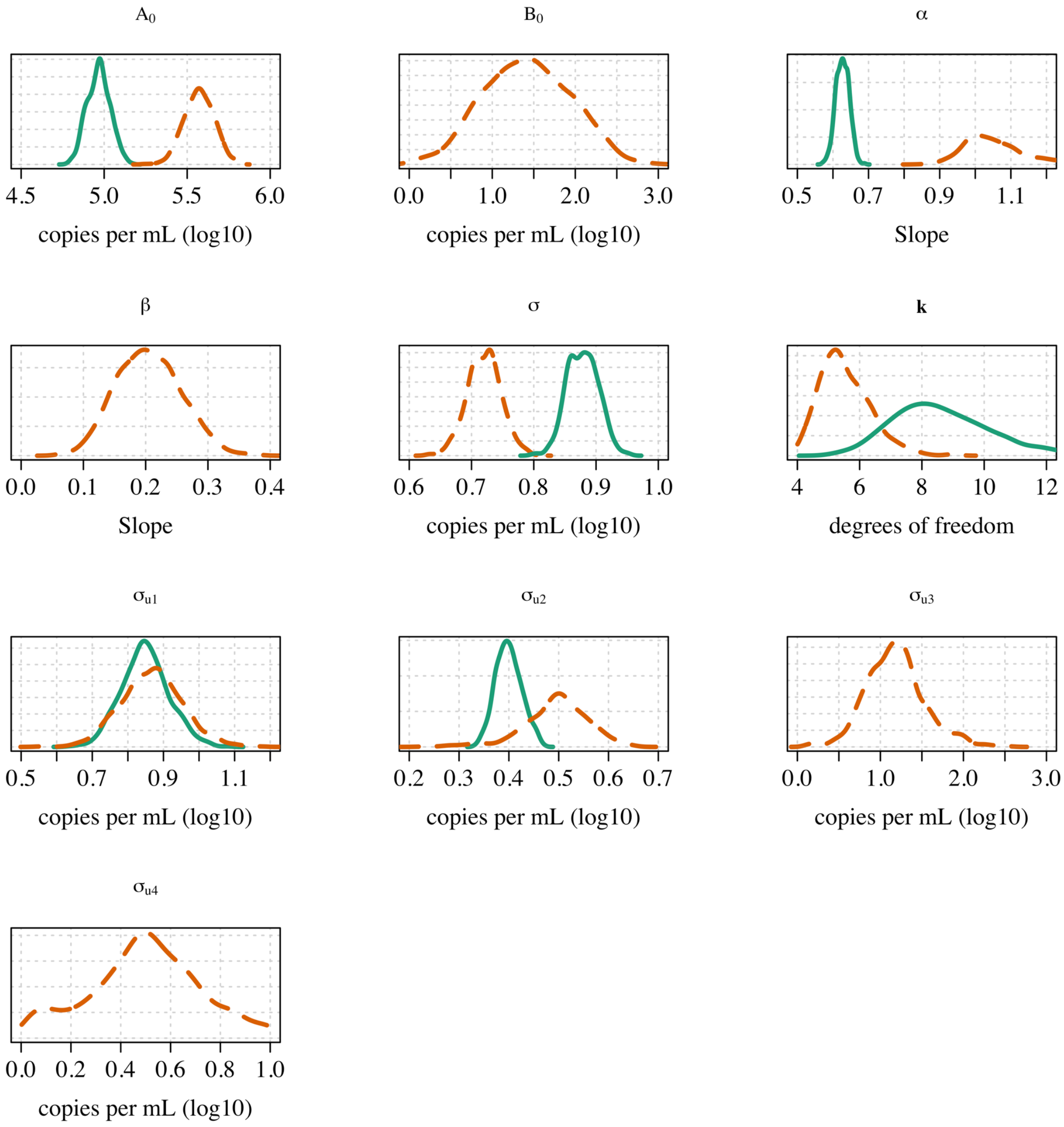

Figure 1 Posterior distributions for the main parameters in the log-linear (thick green lines) and bi-exponential (dashed orange lines) models.

Sensitivity analysis

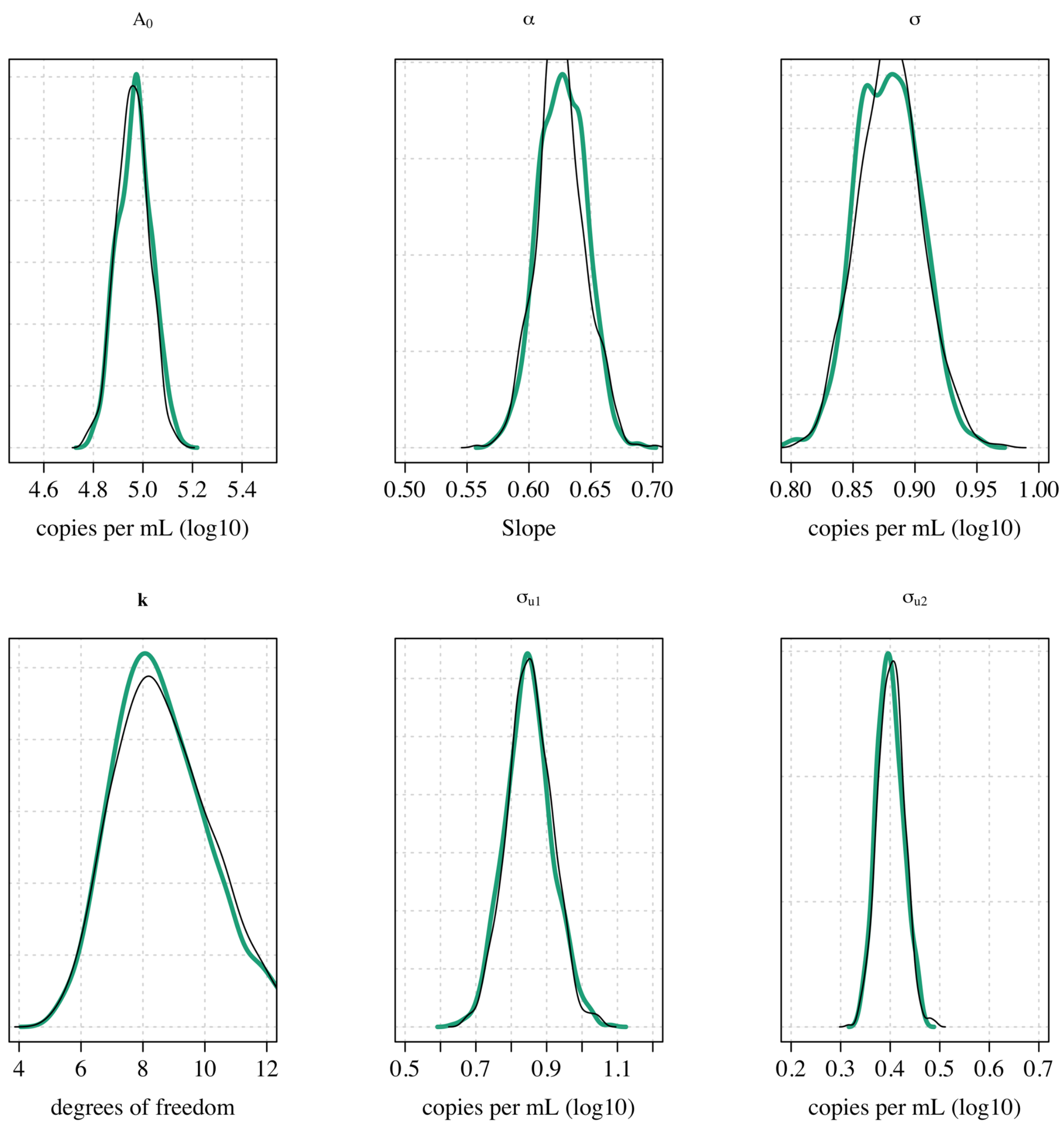

Figure 2 Sensitivity analysis refitting model 1 (log-linear) with quasi-flat priors (all standard deviation values multiplied by 10). Green shows the model fits with the weakly informative priors, black shows the fits with the non-informative priors.

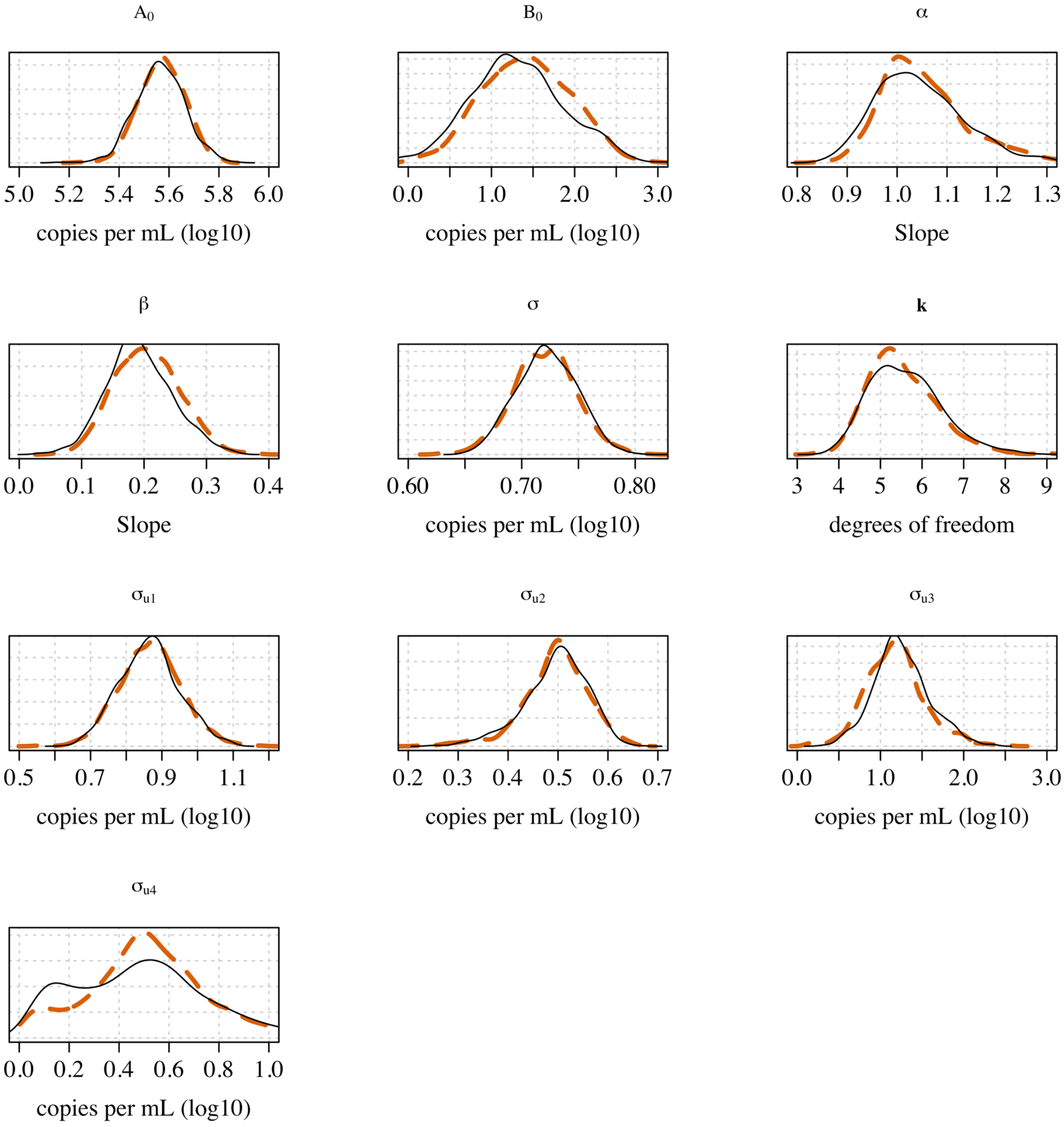

Figure 3 Sensitivity analysis refitting model 2 (bi-exponential) with quasi-flat priors (all standard deviation values multiplied by 10). Dashed orange shows the model fits with the weakly informative priors, black shows the fits with the non-informative priors.

Individual fits

In each plot, the black circles show the individual datapoints; the green line shows the linear model fit (exponential decay); the orange dashed line shows the bi-exponential fit; the purple dotted line shows the additive linear model (spline) fit.
x-axis: days; y-axis: RNA copies per mL.

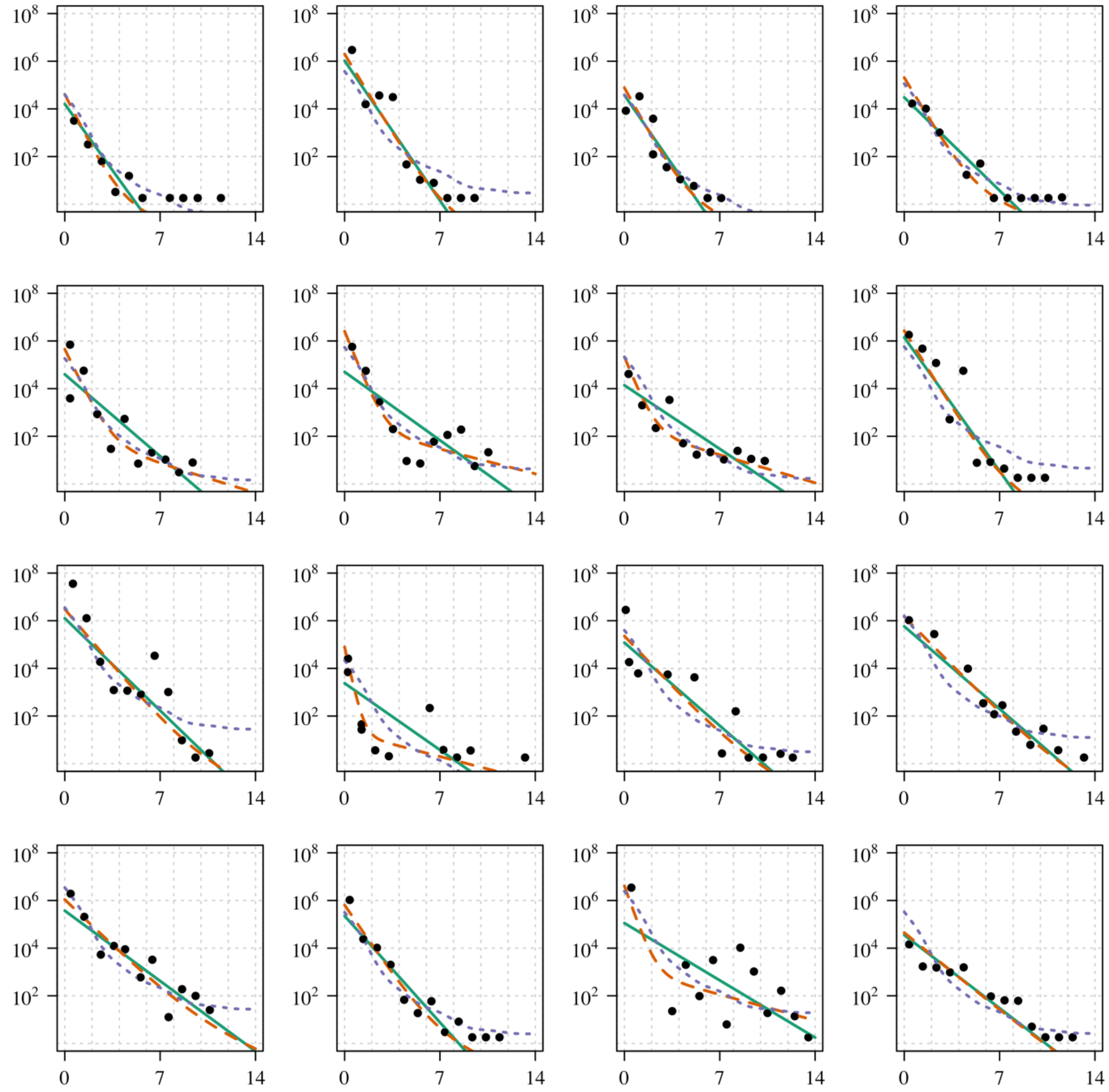

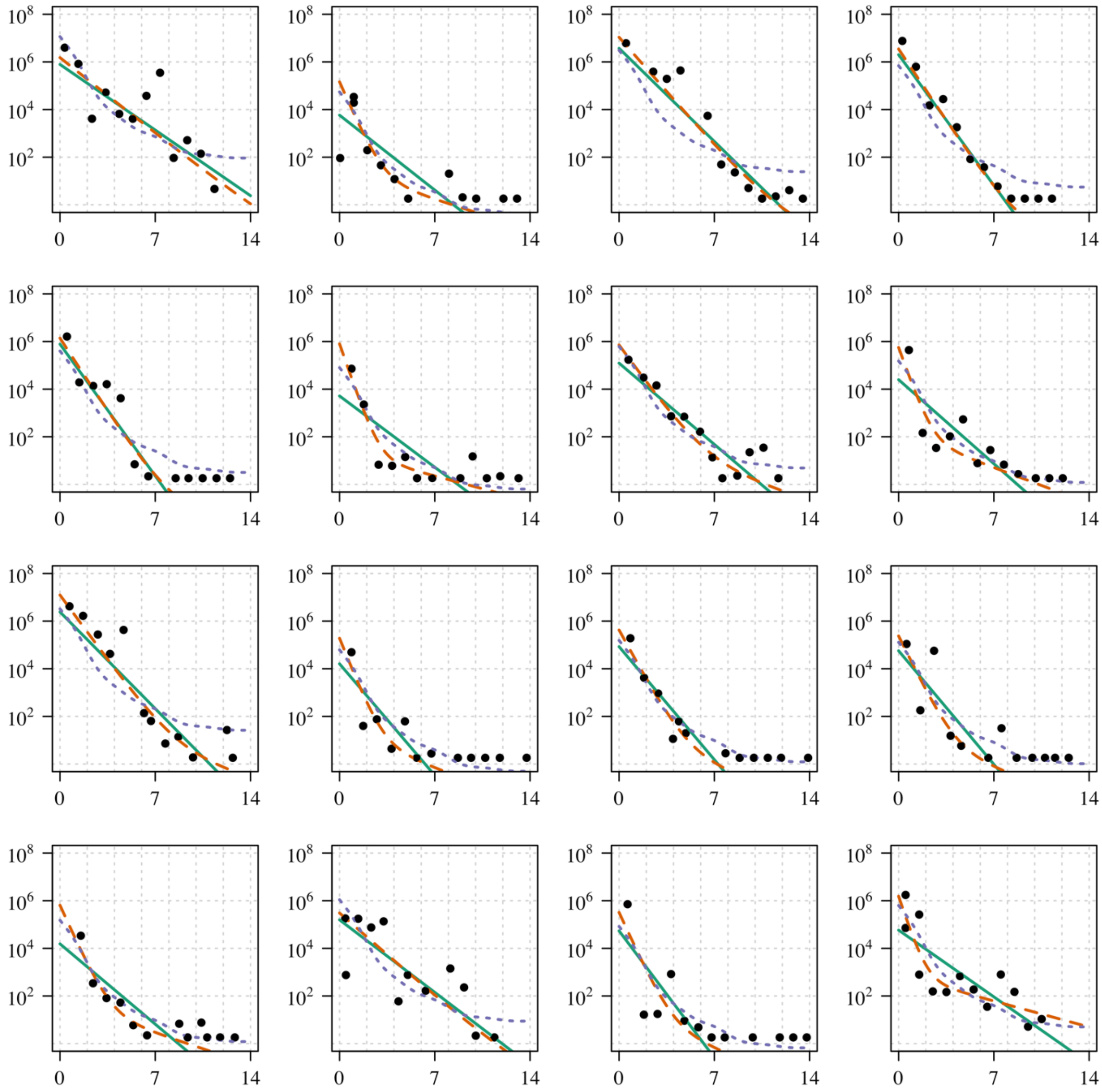
10^8
10^6
10^4
10^2
0
7
14

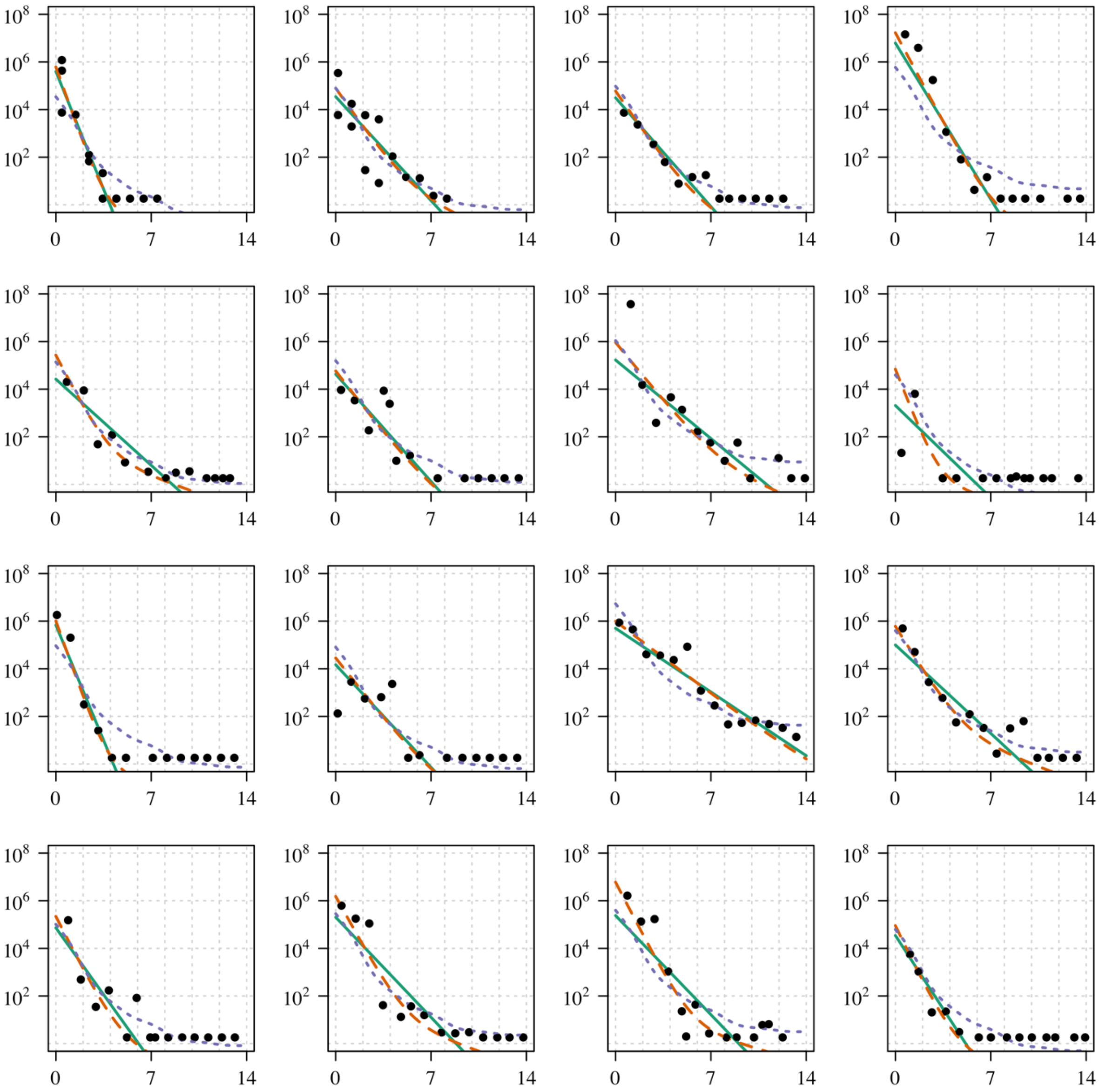

10^8
10^6
10^4
10^2
0
7
14

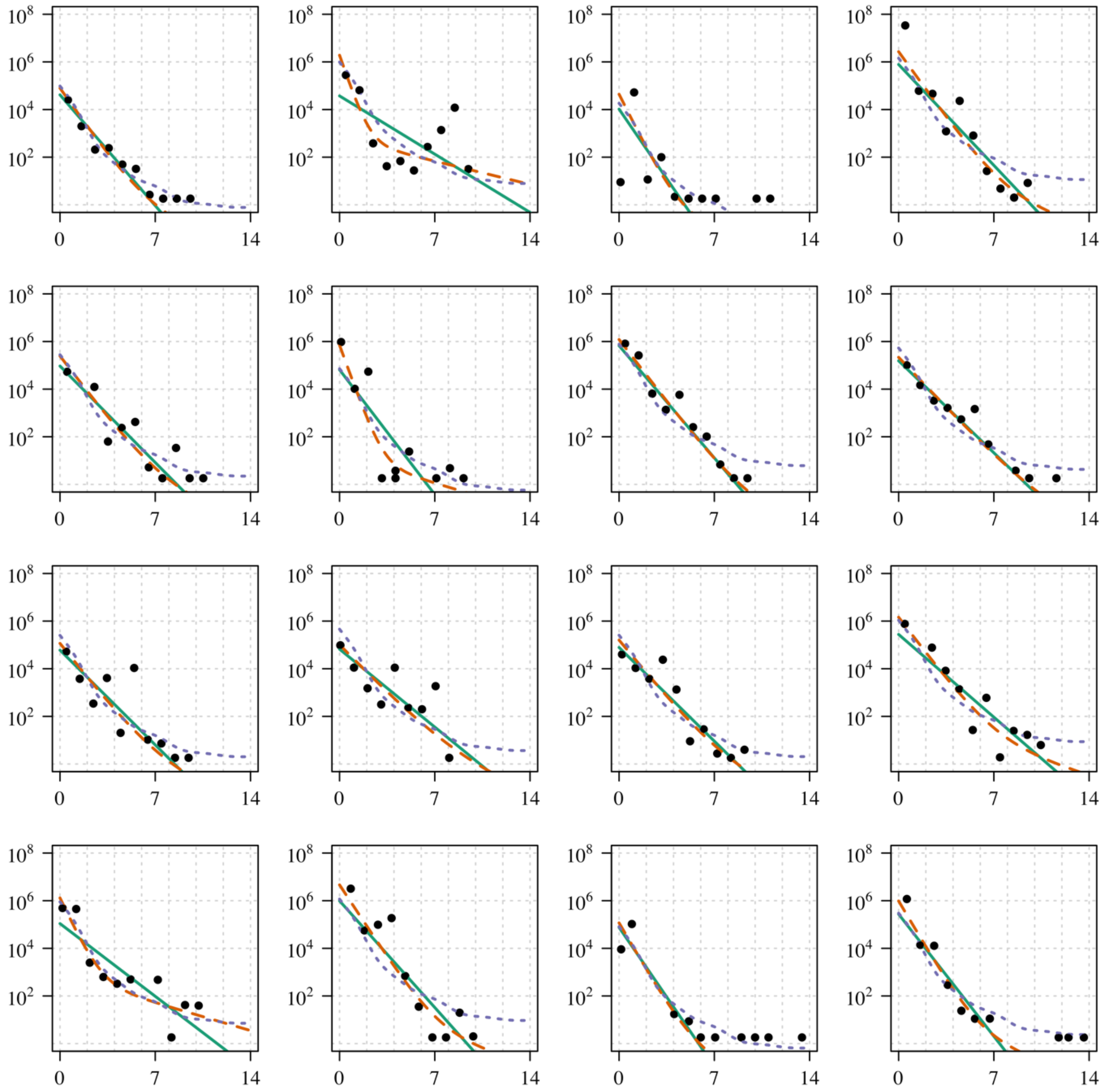

10^8
10^6
10^4
10^2
0
7
14
